# Cast-In-Situ, Large-Sized Monolithic Silica Xerogel Prepared in Aqueous System

**DOI:** 10.3390/molecules23051178

**Published:** 2018-05-15

**Authors:** Wenhui Ding, Xiaodong Wang, Dong Chen, Tiemin Li, Jun Shen

**Affiliations:** Shanghai Key Laboratory of Special Artificial Microstructure Materials and Technology & School of Physics Science and Engineering, Tongji University, Shanghai 200092, China; Giacinta_hui@163.com (W.D.); Dongchen326@163.com (D.C.); ltm1992@163.com (T.L.)

**Keywords:** silica sol, surfactant, binder and pore-forming agent, ambient pressure drying

## Abstract

This paper reports the preparation of cast-in-situ, large-sized monolithic silica xerogels by a two-step acid–base catalyzed approach under ambient pressure drying. Low-cost industrial silica sol and deionized water were used as the silicon source and the solvent, respectively. Hexadecetyltrimethylammonium bromide (CTAB) was used as a modification agent. Different amounts of polyethylene glycol 400 (PEG400) was added as a pore-forming agent. The prepared silica xerogels under ambient pressure drying have a mesoporous structure with a low density of 221 mg·cm^−3^ and a thermal conductivity of 0.0428 W·m^−1^·K^−1^. The low-cost and facile preparation process, as well as the superior performance of the monolithic silica xerogels make it a promising candidate for industrial thermal insulation materials.

## 1. Introduction

SiO_2_ aerogel is a unique nanoporous material with an adjustable density between 3 and 500 mg·cm^−3^, a low thermal conductivity at room temperature, a high porosity (80%~99.8%), a high specific surface area (>1000 m^2^·g^−1^), a high visible-light transmittance, a low refractive index (~1.05), and a modifiable surface chemistry. Therefore, these unique properties facilitate the application of SiO_2_ aerogels in thermal insulation, adsorption, catalytic supports, and so on [1,2,3,4,5].

A large number of efforts have been done to improve the preparation process of the SiO_2_ aerogels, such as the choice of precursors and catalysts, the improvement of the aging process, and the optimization of the surface modification and the drying processes [6,7,8,9,10]. As of yet, SiO_2_ aerogels prepared using tetramethoxysilane (TMOS), tetraethoxysilane (TEOS), and other silicon sources as precursors, as well as ethanol as a solvent via a supercritical drying process [11,12,13] show a superior performance in mechanical property, thermal insulation, and moisture degradation resistance. However, the expensive cost and complex fabrication process hinder their application in industry and civil fields. Recently, cheap and pollution-free water glass and silica sol have been used as precursors to prepare silica aerogels. Song et al. [14]. reported the synthesis of water-glass-based silica aerogels which were dried under ambient pressure. To reduce the shrinkage during the drying process, *N*,*N*-dimethylformamide (DMF) was introduced as a drying control chemical additive (DCCA) to avoid the multiple solvent exchanges. The prepared aerogels achieved an extremely low bulk density of 95 mg·cm^−3^ and a large surface area of 817 m^2^·g^−1^. However, ethanol was still used as a solvent in this method, which was flammable and relatively expensive. This limited its further application for large-scale production. The facile synthesis of monolithic silica aerogels and xerogels with controlled micro/meso/macropores via the sol–gel process has been extensively reported [15,16,17,18,19,20,21,22]. K. Kanamori et al [17]. developed a single-step sol-gel preparation method for transparent hydrophobic methylsilsesquioxane (MSQ) aerogels and xerogels from a single trifunctional precursor, methyltrimethoxysilane. The ambient pressure dried xerogels preserved well pore properties and nanotextures. Based on Kanamori’s work, Shan et al. [19] reported a base-catalyzed one-step synthesis of large-sized monolithic methyltrimethoxysilane-based PMSQ aerogels with deionized water as a solvent, followed by ambient pressure drying. These large-sized monolithic methyltrimethoxysilane-based PMSQ aerogels showed a low density of 75 mg·cm^−3^ and a low thermal conductivity of 0.036 W·m^−1^·K^−1^. Although the PMSQ aerogel was prepared using ambient pressure drying with deionized water as a solvent, methyltrimethoxysilane is still expensive, and the multiple solvent exchange process is relatively time-consuming. In addition, the silica sol can be obtained from water glass by the ion exchange method. In this work, we prepared large-sized monolithic xerogels by a two-step acid—base catalyzed approach under ambient pressure drying, using low-cost industrial silica sol as the silicon source and deionized water as the solvent to reduce the cost of this method. Furthermore, the solvent exchange process is no longer necessary, which simplifies the preparation process.

## 2. Experimental Section

### 2.1. Materials

Silica sol (30 ± 1%, pH = 9.9) derived from water glass was purchased from Zhifa Science&Tech Development Co., Ltd., Wuhan, China. Hexadecetyltrimethylammonium bromide (CTAB, ≥99.0%), hydrochloric acid (HCl, 36.0~38.0%), ammonium hydroxide aqueous solution (NH_3_·H_2_O, 25.0~28.0%), and polyethylene glycol 400 (PEG400, Chemically Pure) were purchased from Sinopharm Chemical Reagent Corp. (Shanghai, China). Deionized water (H_2_O, reverse osmosis) was used in this study.

### 2.2. Sample Preparation

**Figure 1:**
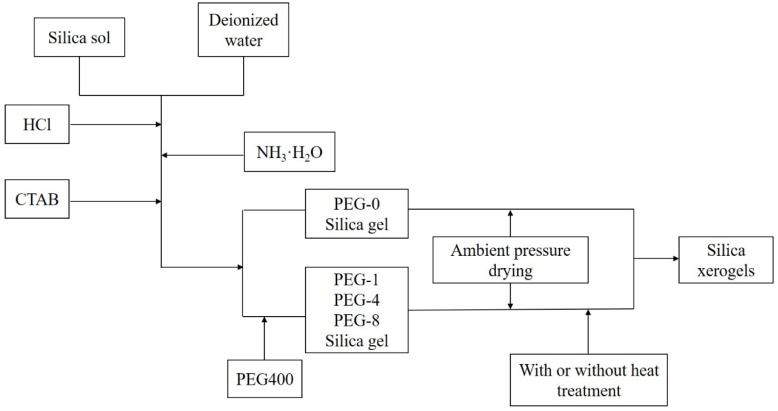
A schematic diagram of the experimental procedure.

The silica gels were prepared via a two-step acid—base catalyzed approach with low-cost industrial silica sol and deionized water as the precursor and the solvent, respectively. A schematic diagram of the experimental procedure is shown in Figure 1. Initially, appropriate amounts of silica sol were dispersed in deionized water and stirred for 5 min at 25 °C, followed by the addition of HCl ([HCl] = 0.08 mol/L) to accelerate the hydrolysis reaction. The resulting solution was then stirred at 25 °C for 1 h. Next, a certain amount of NH_3_·H_2_O ([NH_3_·H_2_O] = 0.15 mol/L) was added to accelerate the condensation reaction. Then, 0.4 g of surfactant CTAB was added, and the mixture was stirred vigorously for 15 min at 40 °C in a water-bath heater. Finally, different amounts of PEG400 were added and stirred for 10 min to obtain different gels. Table 1 details the experimental parameters for the synthesis of the silica gel.

The silica gels were dried at ambient pressure. The solution was firstly transferred into sealed containers and then placed in an oven at 40 °C for 24 h to gel. The gels were further dried in an open container at 50 °C for 48 h to evaporate the solvent slowly. Finally, the gels were heated at 75 °C for 24 h to obtain the silica xerogels. The as-prepared PEG-1, PEG-4, and PEG-8 silica xerogels were calcined at 400 °C for 6 h or calcined at 600 °C for 6 h to obtain heat-treated silica xerogels.

### 2.3. Characterization

The bulk densities ρ of silica aerogels were determined by *ρ* = *M/V*, where *ρ*, *M*, *V* are the measured bulk density, mass and, volume of the silica aerogels, respectively. The morphology of silica xerogels were characterized by a field-emission scanning electron microscope (SEM, Philips XL30FEG, Eindhoven, The Netherland). The Fourier transform infrared (FTIR) spectra of the silica xerogels were performed using a Bruker Tensor 27 spectrometer with the KBr pressed-disk technique. Each spectrum was collected in the range of 400–4000 cm^−1^ with a scanning resolution of 0.5 cm^−1^. The N_2_ adsorption/desorption isotherms were measured by a N_2_ adsorption analyzer (Autosorb-1, Quantachrome, Boynton Beach, FL, USA). The specific surface area and the pore size distribution of the silica xerogels were then calculated from the adsorption branch using the Brunauer–Emmett–Teller (BET) method and the Barrett–Joyner–Halenda (BJH) method, respectively. The thermal conductivity of the silica xerogels was measured at room temperature (25 °C) with a Hot Disk thermal constants analyzer (TPS2500, Gothenburg, Sweden). Each sample was measured three times, and the final results were averaged.

## 3. Results and Discussion

### 3.1. Morphology and Appearance

**Figure 2:**
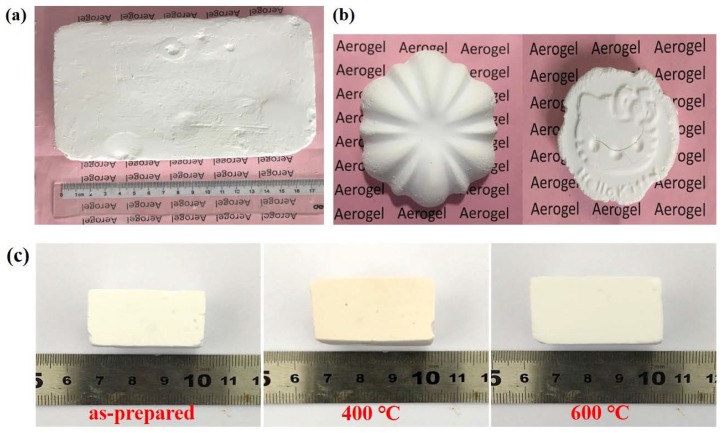
Photographs of typical silica xerogels: (**a**) as-prepared large-sized PEG-1 silica xerogel (17.5 cm × 11 cm × 2.1 cm); (**b**) Cast-in-situ PEG-1 silica xerogel in complex patterns; (**c**) PEG-1 silica xerogel before and after heat treatment at 400 and 600 °C.

Figure 2 shows the image of a typical silica xerogel PEG-1. The as-prepared PEG-1 silica xerogel maintains a completely rectangular shape identical to that of the pattern used for the gel, as shown in Figure 2a. The size of this monolithic xerogel could be further expanded as long as the pattern for gel formation is large enough. Figure 2b presents images of opaque silica xerogels cast in different patterns. As it can be seen from the two figures, silica xerogels with a large size and complex geometry features can be successfully casted, demonstrating a good castability of the silica gels. All of the obtained monolithic silica xerogels with different ratios of PEG400 show a linear shrinkage within ~5%. The macroscopic shapes of the silica xerogels demonstrate almost no change after heat treatment at different temperatures, as shown in Figure 2c. The color of the as-prepared silica xerogels is white and opaque, while the heat-treated silica xerogels at 400 °C are brown. After being further heat-treated at 600 °C, the color of the silica xerogels changed back to white again. This change is due to the residual organic groups in the silica xerogels that firstly carbonized at 400 °C and then decomposed at a higher treating temperature.

**Figure 3:**
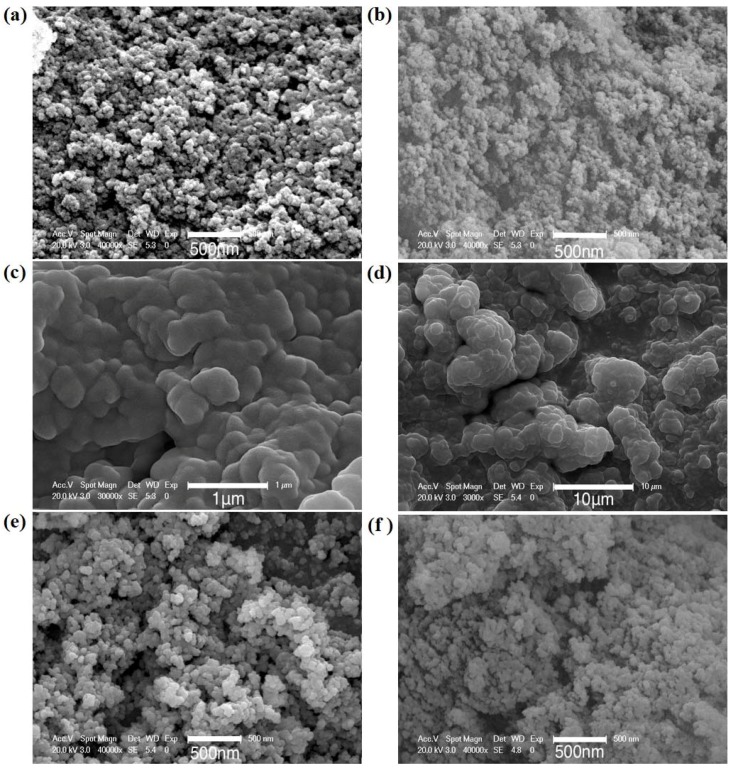
SEM images of silica xerogels (the magnification is labelled in each image): (**a**) as-prepared PEG-0 silica xerogel; (**b**) as-prepared PEG-1 silica xerogel; (**c**) as-prepared PEG-4 silica xerogel; (**d**) as-prepared PEG-8 silica xerogel; (**e**) PEG-8 silica xerogel after 400 °C heat treatment; (**f**) PEG-8 silica xerogel after 600 °C heat treatment.

The SEM images of the monolithic silica xerogels prepared with a different ratio of PEG400 were also taken and shown in Figure 3. As can be seen from Figure 3a–d, the frameworks of as-prepared silica xerogels are three-dimensional network structures composed of irregular spherical particles. The size of the particles increases from 20~50 nm to 1~5 μm with an increasing content of PEG400. The surface morphologies of PEG-8 silica xerogels heat-treated at different temperatures are also shown in Figure 3d–f. As can be seen from the figure, the large particles of PEG-8 silica xerogels turned to small particles after heat treatment at 400 °C, which resulted in a highly porous microstructure of the silica aerogels. After further heat treatment at 600 °C, the particles became even smaller, along with the accumulation of the silica nanoparticles. The evolution of the microstructure was associated with the decomposition of the PEG400 after a high-temperature heat treatment, which indicates that PEG400 plays a role in pore formation. A higher PEG400 concentration causes more silica nanoparticles to accumulate together, thereby leading to the formation of larger particles and pores in the resultant silica xerogels.

### 3.2. Chemical Composition

**Figure 4:**
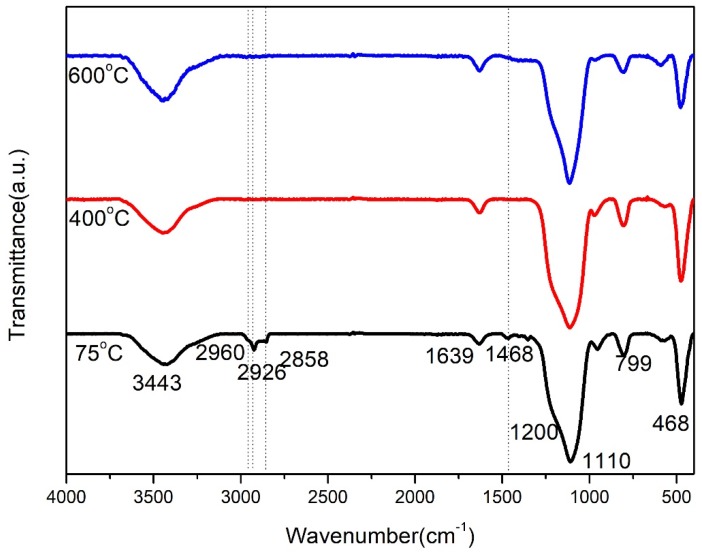
FTIR spectra of PEG-1 silica xerogels heat-treated at different temperatures.

In order to confirm whether the PEG400 was completely decomposed under high-temperature heat treatment, PEG-1 silica xerogels heated at different temperatures were further analyzed by FTIR spectra, as shown in Figure 4. All spectra showed an intense absorption band at around 1110 cm^−1^ and a shoulder at around 1200 cm^−1^ [23], which can be attributed to the asymmetric stretching vibrations of the Si-O-Si bond. The medium intensity band at 468 cm^−1^ and a weak band at 799 cm^−1^ are due to the bending and the symmetric stretching vibrations of the Si-O-Si bond, respectively [13,24,25,26]. The absorption peak at around 3443 cm^−1^ is due to the vibration of hydroxyl group [13]. The peak at around 1639 cm^−1^ is attributed to H-O-H bending vibrations of weakly bound H_2_O adsorbed on the nanoparticle surface of the xerogels [25,27], which overlaps with the absorption peak of the C=O bond. The weak peaks at 2960, 2926, 2858, and 1468 cm^−1^ for the as-prepared PEG-1 silica xerogel are attributed to the vibration modes of the C-H bond [12,13,28], which indicates the existence of the residual carbonaceous organic components in the ambient pressure dried xerogels. After heat treatment at 400 °C, the absorption peaks of C-H bonds become weaker because of the decomposition of residual CTAB and PEG400. After heat treatment at 600 °C, these four peaks vanished, which demonstrates that the organic groups from CTAB and PEG400 in the silica xerogels are entirely eliminated through heat treatment at this temperature. This result is also consistent with the SEM analysis that the PEG400 was removed after heat treatment at 600 °C.

### 3.3. Pore Structure and Specific Surface Area

**Figure 5:**
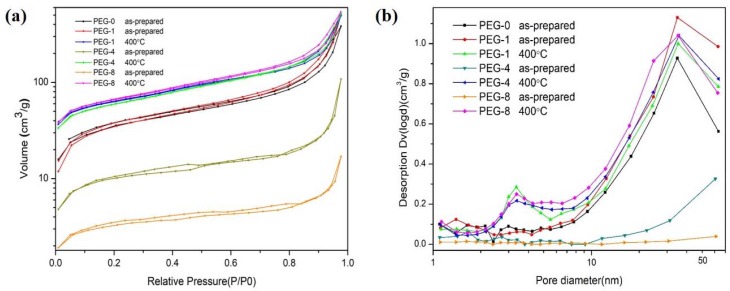
(**a**) Nitrogen absorption/desorption isotherms; (**b**) Pore-size distribution of different silica xerogels.

The pore size distributions of the silica xerogels were determined from the adsorption isotherm using the BJH method, as shown in Figure 5. It is clearly observed in Figure 5a that all the silica xerogels show type IV isotherms with distinct capillary condensation steps, indicating that they are typical mesoporous materials with three-dimensional network structures [29,30]. The linear shrinkage of the silica xerogels were determined by comparing the length of the undried gel with the dried xerogels, or with that after different heat treatments. As listed in Table 2, the linear shrinkage of silica xerogels slightly increases as the heating temperature increases, while the density (*ρ*) of the silica xerogels increases with the amount of PEG400 from 250 to 969 mg·cm^−3^. After heat treatment at 400 °C, the decomposition of PEG400 resulted in a decrease in density in the silica xerogels, an increase in specific surface area from 130 to 220 m^2^·g^−1^, as well as a decrease in average pore size from 24.3 to 14.0 nm (as for PEG-1 silica xerogel). The main reason for these results was that the decomposition of PEG400 increases the proportion of micro- and meso-pores in the samples (as show in Figure 5b), which is also confirmed by the aforementioned SEM and the FTIR analysis. Such changes in pore structure therefore result in an increase in specific surface area and a decrease in average pore size, as shown in Table 2. After further heat treatment at 600 °C, the silica nanoparticles accumulated together, which led to an increase in the average pore size.

### 3.4. Thermal Insulation Behavior

**Figure 6:**
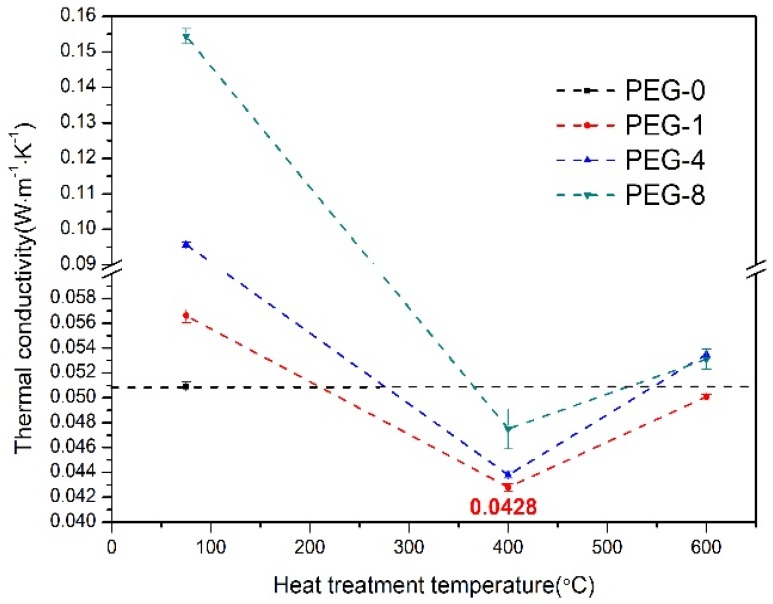
Room-temperature thermal conductivities for the silica xerogels modified with different amount of PEG400 before and after heat treatment at 400 and 600 °C.

The thermal conductivities of different types of silica xerogels were measured and shown in Figure 6. The thermal conductivities of the as-prepared PEG-0, PEG-1, PEG-4, and PEG-8 silica xerogels are 0.0509, 0.0566, 0.0957, and 0.1544 W·m^−1^·K^−1^, respectively. The increasing thermal conductivity with an increasing amount of PEG400 was due to the big clusters formed by PEG400 in the framework of the silica xerogels, which increase the solid thermal conductivity. As known to all, the thermal conductivities of aerogels are made up of three components: thermal transportation by the gas phase, the solid phase, and radiation [2]. The radiative thermal conductivity at ambient temperature contributes little to the total thermal conductivity. In addition, the pore size of the obtained xerogels is mainly in the range of 10–60 nm, which is smaller than the mean free path (~70 nm) of air molecules. Their gas thermal conductivity is thus also largely suppressed. The thermal conductivity of xerogels at an ambient temperature is therefore mainly dependent on the solid thermal conductivity [31]. For the aerogels whose density is higher than 150 mg·cm^−3^, their thermal conductivity mainly depends on the solid thermal conductivity and increases with the increasing density [2]. As shown in our work, the densities of all the prepared silica xerogels are higher than 150 mg·cm^−3^; their thermal conductivities are therefore proportional to the density of the aerogels. As shown in Table 2, the densities of the silica xerogels increase with the amount of PEG400. Therefore, the thermal conductivities of silica xerogels also increase with the amount of PEG400, as shown in Figure 6. We can also observe that the thermal conductivities of all the PEG400-modified silica xerogels decrease after heat treatment at 400 °C. This was related with the decomposition of the PEG400-induced pore structure and the morphological changes that were confirmed by the SEM and the BET analysis. The thermal conductivities of the silica xerogels modified with PEG400 after heat treatment at 400 °C are all lower than those of the as-prepared silica xerogel without PEG400 modification (PEG-0), which indicates that the PEG400 modification could greatly improve the microstructure of the aerogel for better thermal insulation. In addition, we found that the lowest thermal conductivity (0.0428 W·m^−1^·K^−1^) was from the PEG-1 silica xerogel, which means that an appropriate amount of PEG400 was also important for the microstructural control of silica xerogels. Further heat treatment at 600 °C led to the collapse of the porous microstructure and the densification of the silica xerogels. Thus, the thermal conductivities of these PEG400-modified silica xerogels increase slightly after heat treatment at 600 °C compared to those that received heat treatment at 400 °C.

## 4. Conclusions

In summary, we developed a facile strategy for the preparation of low-cost and environmentally friendly cast-in-situ and large-sized monolithic silica xerogels in an aqueous system. The obtained silica xerogels show a linear shrinkage within ~5%. The macroscopic shape of the silica xerogels demonstrate almost no change after heat treatment at high temperatures for 6 h and exhibit a nearly perfect monolithic shape. Different amounts of PEG400 were added as pore-forming agents to create a highly nanoporous microstructure. The PEG-1 silica xerogel treated at 400 °C achieved a minimum thermal conductivity of 0.0428 W·m^−1^·K^−1^ and a density of 223 mg·cm^−3^. Although the thermal conductivity of the obtained silica xerogels was higher than the aerogels prepared via supercritical drying, this thermal conductivity was relatively low among the low-cost thermal insulation materials derived via the ambient pressure drying process. The resulting low-cost silica xerogels with a low density and a comparatively high thermal insulation performance are very suitable for building thermal insulation and other areas requiring improved thermal efficiency.

## Figures and Tables

**Table 1:** Experimental parameters for the synthesis of the silica gel.

Samples	Silica sol/mL	Deionized Water/mL	HCl (0.15mol/L)/mL	NH_3_·H_2_O (0.08mol/L)/mL	CTAB/g	PEG400/mL
PEG-0	10	20	10	10	0.4	0
PEG-1	10	20	10	10	0.4	1
PEG-4	10	20	10	10	0.4	4
PEG-8	10	20	10	10	0.4	8

**Table 2:** Parameters of different silica xerogels. (ap: as-prepared).

Samples	*ρ* (mg·cm^−3^)			Specific Surface Area (m^2^·g^−1^)			Average Pore Size (nm)			Linear Shrinkage (%)		
	ap	400 °C	600 °C	ap	400 °C	600 °C	ap	400 °C	600 °C	ap	400 °C	600 °C
PEG-0	250	—	—	127	—	—	18.7	—	—	2.9	—	—
PEG-1	302	223	221	130	220	233	24.3	14	15.8	1.9	2.3	2.9
PEG-4	624	281	273	35	212	170	19.3	15.1	19.5	1.9	2.5	3.3
PEG-8	969	271	266	19	232	143	9.6	14.4	19.1	3.5	4.1	5
